# Fecal microbiota transplantation – where are we?

**DOI:** 10.3325/cmj.2021.62.52

**Published:** 2021-02

**Authors:** Ivana Mikolašević, Goran Hauser, Maja Abram, Tajana Filipec, Kanižaj, Marija Radić, Irena Krznarić, Zrnić

**Affiliations:** 1 Department of Gastroenterology, Clinical Hospital Center Rijeka, Rijeka, Croatia; 2 Department of Gastroenterology, Merkur University Hospital, Zagreb, Croatia; 3 Faculty of Medicine, University of Rijeka, Rijeka, Croatia ivana.mikolasevic@gmail.com; 4 Faculty of Health Studies, University of Rijeka, Rijeka, Croatia; 5 Department of Microbiology and Parasitology, University of Rijeka, Faculty of Medicine, Rijeka, Croatia; 6 Zagreb University School of Medicine, Zagreb, Croatia

In addition to its antimicrobial protective role, the gut microbiota affects human metabolism and immunity as well as inflammatory and neuro-hormonal responses. Due to its beneficial effects on health even up to the genetic level, it is referred to as a “forgotten organ,” “virtual organ,” or “other brain” (1). The human gut microbiome differs among individuals and usually does not change over time. Its composition is affected by several environmental factors (1-3). Fecal microbiota transplantation (FMT) or bacteriotherapy (health donor stool transplantation) is the instillation of a fecal suspension taken from a healthy donor into the upper or lower gastrointestinal (GI) tract of a patient, with an aim to enrich and normalize his or her gut microbiota. Although the method has been known for centuries, interest in it has significantly grown over the last few decades. The two main reasons are the global epidemic of *Clostridioides difficile* (CDI) infection and an improved knowledge of the GI microbiome and its involvement in various conditions. It seems that FMT may also transfer host phenotype.

Many studies have shown the effectiveness of FMT when used as a therapy for recurrent and refractory CDI. Excellent treatment results were also observed in patients with multiple diseases and in immunosuppressed patients. The success rate was exceptional, up to 90%, in recurrent or refractory CDI (4,5). According to the known clinical evidence, no absolute contraindication for FMT has been encountered (6). Besides the use in CDI patients, the implementation of FMT would probably be expanded by new insights linking gut microbes to the pathophysiology of other intestinal and extraintestinal diseases.

Stool donor may be any health individual, such as partner, relative, friend, or genetically unrelated and formerly unknown healthy person. Donors should be tested for various infections and conditions that carry an increased risk of disease transmission (5,6).

Regarding stool preparation, a freshly prepared donor stool specimen should be transferred within 6 h after evacuation. Normal saline is mostly used, but water or milk are also viable alternatives. Solid stool is usually diluted in solvent at a ratio of 1:3 or 1:5. The aim is to prolong the duration of the transferred stool in the recipient's gut, so it is important to ensure a high viscosity of the stool suspension. Stool specimen is homogenized and then filtered. A processed specimen is usually instantly infused into the gastrointestinal tract, but it can also be prepared for freezing or for the production of encapsulated preparations (7,8). Mastering the preparation process of frozen stool is crucial for the establishment of a stool bank, which would ensure accessibility without waiting for new donations or their screening. Randomized control trials found no difference between the use of fresh and frozen fecal samples for the treatment of resistant CDI (7-9).

Fecal transfer can be performed in several ways. In the colonoscopic method, fecal suspension is infused trough a working channel of colonoscope and installed in the colon ascendens and cecum. In most cases, the volume of the installed stool is about 200-500 mL (6,10). Another method is the application by means of nasojejunal tube. In this case, the volume of fecal suspension is much lower (25-50 mL). Despite all the benefits, long-term outcomes and the potential adverse events (AEs) related to FMT remain the main concerns. AEs are usually divided into two categories: microbiota-related and delivery-related (11). Microbiota-related AEs result from the interaction between transplanted microbiota and the host (fever, bacteremia, allergic reaction, disease exacerbation or relapse, and transmission of unwanted pathogenic organism infection). Delivery-related AEs are a consequence of the modality of infusion (vomiting, aspiration pneumonia, post-procedural abdominal pain, nausea, proctalgia or anorectal discomfort, bowel perforations, and sore throat) (11).

## FECAL TRANSPLANTATION FOR INDICATIONS OTHER THAN *C. DIFFICILE*

### Irritable bowel syndrome

The loss of gut microbiota balance, called dysbiosis, leads to a loss of intestinal homeostasis and ultimately to the occurrence of certain intestinal and extraintestinal disorders, including irritable bowel syndrome (IBS) (12). In IBS, abdominal pain is often accompanied by a change in stool rhythm. According to recent evidence, intestinal microorganisms play a key role in the onset and development of this disorder (13). The idea that the microbiota causes the IBS symptoms is not new. Therefore, several treatment modalities involve intestinal microbiota modulation, either by using probiotics or, more recently, by using FMT. Unfortunately, the results are not uniform, and sometimes it is difficult to differentiate the cause from the consequence. In addition, no single hypothesis is applicable to all patients. Initial data showed questionable results of IBS treatment with FMT, but there is an obvious trend of positive results, which can be attributed to proper donor selection. Several clinical studies reported the effectiveness of FMT in IBS treatment, but with ambiguous results.

Huang et al (14) performed FMT on 30 Chinese patients with refractory IBS. After baseline evaluation and one month after FMT, they extracted genomic DNA from fecal samples. Clinical efficacy and safety were monitored for six months. FMT improved almost all clinical disease indices and reduced the high depression and anxiety level typical for patients with IBS. FMT responders showed an undoubtedly higher Shannon diversity index than non-responders. Furthermore, a good safety profile was obtained.

A group of Finnish authors (15), who performed single-infusion FMT during colonoscopy in IBS patients, compared autologous and allogenic FMT. Unfortunately, symptom reduction was not significant in any group. This could be attributed to the fact that patients were not divided into IBS subtypes, as FMT might not be an optimal treatment method in unselected patients with all IBS subtypes (15). This was also confirmed in a meta-analysis (16) that included four studies.

Somewhat more positive results were obtained by a meta-analysis of five randomized controlled trials (RTC) (17) recruiting mostly IBS patients (>90%). The results showed that FTM in capsules was not more effective than placebo. On the other hand, in two pooled RCTs, FMT taken from colonoscopically infused donor stool showed better results than that from the autologous stool. One study involving FMT from donor stool infused with the help of nasojejunal tube suggested a slight beneficial trend in comparison with autologous stool application (17). However, no firm conclusions can yet be drawn because this meta-analysis mostly included studies of poor quality.

A step forward was a uniquely designed Norwegian study (18). All patients received stool from a super donor, ie, an individual who had met well-defined and described criteria. An additional advantage of the study was the number of patients involved (N = 165) and the administration of two doses of stool (30 and 60 g) in the upper intestinal tract. In addition to clinical indicators of IBS, patients were also monitored for dysbiosis index, and 16S rRNA gene sequencing was used to monitor intestinal bacterial profile one month after FMT. The clinical response was very good, especially in patients who received 60 g of fresh frozen stool. Thus, the stool dose required was relatively well defined. In addition to the clinical response, the intestinal bacterial profile also changed, indicating that the change in the symptoms was associated with the change in the bacterial composition in the intestine. According to the authors, donor selection was crucial for FMT success in IBS (18).

Conclusively, FMT is a useful therapeutic method in some patients with IBS, but is not a universal remedy. With the new generation of sequencing and bioinformatics, we can detect microbiota changes and obtain characteristic profiles of healthy and sick people. The research goal is a clearly defined microbiological composition of the donor stool, allowing targeted pairing with patients who have a specific microbial deficit. Of course, the chances of the method's success can be increased by a specific diet (low-FODMAP diets), which enhances the growth of desirable bacteria and suppresses the growth of harmful bacteria.

### Inflammatory bowel disease

In recent years, FMT has been studied as a promising therapeutic approach for patients suffering from inflammatory bowel disease (IBD). IBD is suggested to develop as a result of synergy/interaction of genetic susceptibility and environmental factors (IBD patients have a reduced microbial composition, diversity, and richness), which leads to inappropriate intestinal immune activation through a weakened intestinal barrier (19). This results in clinical and endoscopic findings characteristic of IBD patients (19). Current therapeutic options for IBD are mainly based on pharmacological approaches and include traditional or biological medications that control inflammation and maintain remission (19). In the absence of curative treatment options, modulation of gut microbiota by FMT has been suggested as a promising strategy.

Motivated by the excellent results of FMT for the treatment of CDI many authors have regarded FMT with enthusiasm as a possible treatment option for other bowel diseases. FMT is a non-immunosuppressive approach that addresses the microbial dysbiosis underlying the IBD pathogenesis. The beneficial FMT effect has been more investigated in the context of ulcerative colitis (UC) than in the context of Crohn's disease (CD) (20). Several case-series, cohort studies, and four RCT investigated the usefulness of FMT in UC treatment (21-26). Furthermore, a meta-analysis on UC and FMT was published (26). This meta-analysis included 24 cohort studies with 307 UC patients treated with FMT (25). A pooled proportion of IBD patients that attained clinical remission was 33%. The study also included four RTCs recruiting 140 patients treated with FMT. The results showed a significant association between FMT and achievement of clinical remission (25). Additionally, when the authors excluded the smallest study (26), an even higher association was observed (25). Namely, this study, as opposed to the other three, involved the administration of only two infusions to the upper gastrointestinal tract (25,26). The meta-analysis authors stressed that multiple infusions and lower GI administration might be associated with a higher incidence of remission in UC patients. According to data, factors that can influence FMT efficacy in patients with IBD are the route of delivery, pre-treatment antibiotics, anaerobic FMT preparation, the severity of IBD, dosing, donor microbial/metabolic profile, recipient microbial/metabolic profile, diet, and single vs multi-donor use. Some unknown facts, such as the dosing regimen and optimal route of administration, need to be further investigated (25). In the context of IBD, the appropriate donor selection remains a challenging issue, since individual donor characteristics are suggested to be associated with FMT efficiency in IBD. It is assumed that it is better to use a stool specimen from an unrelated than from a related donor in order to avoid shared genetics and environmental determinants from the donor GI tract. Donor characteristic such as age, diet, and microbial profile further affect the FMT success in IBD. Finally, the use of multi-donors is advised in order to achieve functional diversity (25). Unlike in CDI, it is unclear which type of stool (fresh vs frozen) is better to apply in IBD patients (25).

The data on FMT in CD are limited, without powerful RCTs. The major issue is that CD displays considerable heterogeneity in disease distribution and clinical phenotypes. Because of this, each clinical phenotype is probably associated with a different response to FMT treatment (25,26).

Taken together, therapeutic gut microbiota manipulation with FMT in IBD patients is a thrilling and rapidly developing issue in gastroenterology. Available data of the beneficial impact of FMT on remission induction in UC patients are encouraging. However, many issues are unknown, such as the use of FMT as a maintenance approach in UC or the role of FMI in the context of CD. As mentioned, all available studies have investigated the favorable outcome of FMT in achieving remission in UC, with little or no data on the role of FMT in remission maintenance. Additionally, there is a need for longitudinal studies on FMT efficacy, durability, and safety in patients with IBD that could help us to improve our knowledge and facilitate FMT personalization (25,26).

## **FECAL MICROBIOTA TRANSPLANTATION** **FOR EXTRAINTESTINAL DISEASES**

There is growing data suggesting an important role of gut microbiota in the pathophysiological pathways of many extra-intestinal diseases.

### Metabolic disease

Today, we are faced with an epidemic of obesity, type 2 diabetes (T2D), and metabolic syndrome, all of which are closely related to nonalcoholic fatty liver disease (NAFLD). NAFLD is the commonest form of chronic liver disease. Its incidence is growing in parallel with the increasing incidence of the mentioned metabolic disorders. A growing number of data support the hypothesis that gut microbiota is closely associated with MetS and its related conditions. Recent data have suggested that microbiota-dependent changes of gut epithelial permeability, production of bile acid, and systemic immune response are related to MetS pathogenesis (27). Thus, the usefulness of FMT in the treatment of MetS and its associated conditions have attracted research interest, although there are limited data in humans. For example, Vrieze et al (28) investigated the effects of infusing either autologous gut microbiota or gut microbiota from healthy thin donors to 18 receivers with MetS on microbiota composition and glucose metabolism. The authors observed an amelioration of receivers’ insulin resistance (IR) six weeks after microbiota infusion (28). Furthermore, there is only one small study regarding the use of FMT in NAFLD patients involving 21 patients (29). The authors assessed whether FMT that used stool from a lean, healthy donor applied to NAFLD patients with MetS would improve IR six weeks after FMT, hepatic proton density fat fraction (PDFF) obtained by MR elastography at six months, and intestinal permeability six weeks after FMT (29). Fifteen NAFLD patients received an allogenic FMT, while 6 received autologous FMT (29). The two groups did not significantly differ in HOMA-IR score and liver PDFF (29). On the other hand, allogenic FMT NAFLD patients with increased permeability of the small intestinal at the beginning of the study had a compelling reduction six weeks after the allogenic procedure (29). Another interesting observation in this study was that the NAFLD patients who experienced an improvement in intestinal permeability had a higher fecal microbiota heterogeneity (29). These observations are encouraging in terms of the permeability of the small intestinal as a consequence of allogenic FMT (29). Furthermore, authors did not find a positive effect of FMT on MR elastography findings in the follow-up period (29). This could be related to the number of FMT procedures, because the effect of only one procedure might not persist during the six months following FMT (29). Thus, the question is if repeated FMT would be able to prevent the gut microbiota to reverse to baseline (29). However, given the sample size of this study, further larger studies are needed.

### Neuropsychiatric conditions

Healthy gut microbiota is the key factor sustaining the functional stability of the gut-brain axis (30). Some factors can influence gut microbiota homeostasis (such as excessive reproduction of pathogenic bacteria), which is connected to gut-brain axis disturbance. This eventually can be related to various neurological and psychological disorders. Namely, the gut microbiome has a major role in neuroendocrine, neural, and immune pathways (31). The most important pathway is the brain-gut-microbiota axis. The GI tract microbiota may engage a bidirectional communication network in order to adjust the brain's function and development, and finally behavior (30-32). Alternations in the microbiota diversity, as an outcome of aging, mediate disorders of the central nervous system (CNS), particularly those often appearing in the elderly, such as Parkinson’s syndrome and Alzheimer's disease. Microbiome is also a potential diagnostic and therapeutic target in other non-degenerative CNS diseases, such as stroke, and even in drug addiction therapy (30). Additionally, growing data suggest that gut dysbiosis affects mental health and psychiatric diseases (such as bipolar disorder, autism spectrum disorder, depression, obsessive-compulsive disorder, anxiety, schizophrenia) through the gut-brain axis (30-32). Consequently, FMT was suggested as a method to reconstruct gut microbiota and therefore treat many neurological and psychiatric diseases related to the gut-brain axis (30,31). It is believed that gut microbiota modulation may also affect the pharmacokinetics of some medications used for patients with neurological conditions, leading to a higher efficacy of standard medical treatment for these disorders (30). The importance of a healthy-donor FMT was most evident in patients with autism spectrum disorder (30), who experienced decreased symptoms intensity. Additionally, several animal studies and some case reports in humans suggested a positive role of FMT in the treatment of Parkinson’s syndrome, multiple sclerosis, Alzheimer's disease, and stroke. Hypothetically, FMT can decrease the infarct size in patients with stroke by decreasing the immune response and blocking the pro-inflammatory immune cells trafficked to the infarct area (30). Further studies on this topic would be of a great clinical interest (30).

Despite the promising results, the data supporting the beneficial effect of FMT in neuropsychiatric disorders are confined to case reports, case series, and one observational study. Thus, large, randomized investigations are needed to delineate the role of FMT in the treatment of neuropsychiatric conditions (30-32). Nowadays, there are many ongoing trials on FMT in the context of neuropsychiatric disorders. expanding the amount of evidence on the beneficial effect of gut microbiota modulation by FMT in neuropsychiatric disorders.

### Hematologic diseases

The available data concerning the beneficial effect of FMT for the treatment of hematologic disorders are very sparse. An interesting pilot study by Kakihana et al (33) assessed the safety of FMT in stem cell transplantation. They performed FMT in four patients with acute graft-vs-host disease. Of 4 included patients, 3 had a complete clinical response after FMT treatment, while 1 patient had a partial response. Interestingly, patients with a complete response following FMT had a domination of beneficial bacteria (*Lactobacillus, Bifidobacterium, Bacteroides,* and *Faecalibacterium*). This study is important as it confirmed the safety of the use of FMT in immunocompromised patients.

### Chronic hepatitis B infection

Hepatotropic viruses, namely chronic hepatitis B (HBV), are a considerable etiological factor for the development of cirrhosis and hepatocellular carcinoma. Treatment options of chronic HBV infection include nucleotide inhibitors and pegylated interferon. However, treatment is successful in only a minority of individuals (32,34). The decrease in gut microbiota diversity and dysbiosis in patients with hepatitis B (32,34) sparked an interest in gut microbiota modulation in these patients. Ren et al (34) analyzed 18 patients with chronic HBV infection who were on antiviral therapy. Five patients received FMT and 13 patients continued with the antiviral treatment. Patients treated with FMT experienced gut microbiota changes connected with a significant decline in HBeAg titer. HBeAg titer was further decreased following an additional FMT. Remarkably, 2 HBV patients attained HBeAg clearance at the end of the monitoring. On the other hand, none of HBV patients receiving chronic antiviral treatment had HBeAg clearance (34). This study shows that FMT is promising in the treatment of chronic HBV infection, warranting further investigations, especially randomized control trials.

## CONCLUSION

Complex and dynamic intestinal microbiota is seen as a crucial factor affecting human health. Alternations in bacterial community observed in many diseases provide us with an opportunity to investigate and analyze new therapeutic methods. FMT is a generally safe therapeutic procedure, but similarly to all other therapeutical agents, it has potential adverse effects. The most frequently declared side effects were nausea/vomiting, bloating, abdominal discomfort, and diarrhea. FMT efficacy as a therapeutic method in different diseases warrants further research. Lately, many clinical trials and case reports showed FMT to be a useful primary therapeutic method in difficult-to-treat diseases, such as UC, IBS, pouchitis, obesity, neuropsychiatric conditions, IR, etc. Nevertheless, response rates were not as remarkable as when FMT was used for CDI. While these data are encouraging and confirm the concept to some extent, available investigations do not clearly support a more frequent FMT implementation. However, adjusted policy of FMT use, step-up, or intensive-dosing multi-donor FMT in IBD patients achieved an excellent treatment reaction. Thus, FMT should be improved in other indications beside CDI. In the near future, it will be possible to test the recipients and donors before FMT. This approach could allow the matching of patients with optimal stool donors for each indication in a form of patient-based medicine (32).
